# Clinical impact of *Achromobacter xylosoxidans* colonization/infection in patients with cystic fibrosis

**DOI:** 10.1590/1414-431X20155097

**Published:** 2016-02-23

**Authors:** M.C. Firmida, R.H.V. Pereira, E.A.S.R. Silva, E.A. Marques, A.J. Lopes

**Affiliations:** 1 Programa de Pós-Graduação em Ciências Médicas, Universidade do Estado do Rio de Janeiro, Rio de Janeiro, RJ, Brasil; 2 Departamento de Microbiologia, Imunologia e Parasitologia, Faculdade de Ciências Médicas, Universidade do Estado do Rio de Janeiro, Rio de Janeiro, RJ, Brasil; 3 Laboratório de Bacteriologia, Hospital Universitário Pedro Ernesto, Universidade do Estado do Rio de Janeiro, Rio de Janeiro, RJ, Brasil

**Keywords:** Cystic fibrosis, *Achromobacter* spp., Achromobacter xylosoxidans, Microbiology

## Abstract

The rate of diagnosis of colonization/infection of the airways with *Achromobacter xylosoxidans* has increased in cystic fibrosis patients, but its clinical significance is still controversial. This retrospective, case-control study aimed to evaluate the clinical impact of *A. xylosoxidans* colonization/infection in cystic fibrosis patients. Individuals who were chronically colonized/infected (n=10), intermittently colonized/infected (n=15), and never colonized/infected with *A*. *xylosoxidans* (n=18) were retrospectively evaluated during two periods that were 2 years apart. Demographic characteristics, clinical data, lung function, and chronic bacterial co-colonization data were evaluated. Of the total study population, 87% were pediatric patients and 65.1% were female. Individuals chronically colonized/infected with *A. xylosoxidans* had decreased forced expiratory volume in 1 s (51.7% in the chronic colonization/infection group *vs* 82.7% in the intermittent colonization/infection group *vs* 76% in the never colonized/infected group). Compared with the other two groups, the rate of co-colonization with methicillin-resistant *Staphylococcus aureus* was higher in individuals chronically colonized/infected with *A*. *xylosoxidans* (P=0.002). Changes in lung function over 2 years in the three groups were not significant, although a trend toward a greater decrease in lung function was observed in the chronically colonized/infected group. Compared with the other two groups, there was a greater number of annual hospitalizations in patients chronically colonized/infected with *A. xylosoxidans* (P=0.033). In cystic fibrosis patients, there was an increased frequency of *A. xylosoxidans* colonization/infection in children, and lung function was reduced in patients who were chronically colonized/infected with *A. xylosoxidans*. Additionally, there were no differences in clinical outcomes during the 2-year period, except for an increased number of hospitalizations in patients with *A. xylosoxidans*.

## Introduction

The genus *Achromobacter* contains genetically distinct species and subspecies, and has not been fully characterized (1 2 3 4 15). *Achromobacter* spp. are Gram-negative, aerobic, nonfermenters of glucose bacilli that are widely distributed in the environment. *Achromobacter xylosoxidans* is the most common bacillus in clinical samples and is recognized as an emerging and multidrug-resistant microorganism that causes various opportunistic infections and nosocomial outbreaks (3,6). Most knowledge on *A. xylosoxidans* has been obtained from studies on populations living in regions where cystic fibrosis (CF) is prevalent (3,6).

The rate of colonization/infection with *A. xylosoxidans* in individuals with CF varies between 2% and 17.9% (7,8) and is increasing worldwide. However, this frequency may be underestimated because this organism can be confused with *Pseudomonas aeruginosa*, bacteria from the *Burkholderia cepacia* complex (BCC), and *Stenotrophomonas maltophilia*, particularly in laboratories that are not specialized for evaluation of CF (9).

The factors that predispose patients to colonization/infection have not been fully determined. Frequent exposure to antibiotics, particularly during treatment for chronic colonization with *P. aeruginosa*, may favor the emergence of this and other Gram-negative, multidrug-resistant bacteria (10,11). The possibility of person-to-person transmission, the association of *A. xylosoxidans* colonization/infection with pulmonary inflammation, and an increased frequency of exacerbations have been demonstrated. However, the clinical impact of colonization/infection of *A. xylosoxidans* in CF patients is still controversial (6,15). Therefore, the present study aimed to evaluate the clinical impact of *A. xylosoxidans* colonization/infection in patients with CF.

## Material and Methods

### Study design

This retrospective, case-control study evaluated patients with a confirmed diagnosis of CF (16). These patients were regularly monitored at the Instituto Fernandes Figueira, Fundação Oswaldo Cruz and Policlínica Piquet Carneiro, Universidade do Estado do Rio de Janeiro (Brazil). Patients' respiratory secretion culture results were obtained between January 2003 and December 2011 at the Laboratório de Bacteriologia, Hospital Universitário Pedro Ernesto (LBACT-UERJ).

The protocol conformed to the World Medical Association Declaration of Helsinki and was approved by the Research Ethics Committee of the Universidade do Estado do Rio de Janeiro (No. CAAE: 00716512.0.3001.5269).

### Patients

A total of 238 individuals (155 females and 83 males) with CF were regularly monitored in these referral centers, of whom 25% were adults (≥18 years). The routine follow-up period consisted of quarterly consultations, except for infants, who were monitored monthly. The interval between consultations was shortened depending on clinical need. At each visit, the general medical condition, weight, height, and lung function of patients were evaluated; and respiratory secretions were obtained for culture (sputum or oropharyngeal swab for non-expectorating children). All material obtained at these centers was sent to the LBACT-UERJ. In this institution, cultures of respiratory secretions were carried out according to standardized protocols established for CF patients. Cultures were performed every 3 months throughout the study (17).

### Identification of *Achromobacter*

#### Phenotypic methods

Isolates that were identified as *Achromobacter* spp. by the Vitek 2 Compact system using Gram-negative cards (reference no. 21341; bioMérieux, France) were subjected to further identification via a large panel of phenotypic tests, as previously described (18,19).

#### Molecular methods

To identify each isolate, DNA was extracted by the boiling lysis method, and the entire *16S* rRNA gene was amplified by PCR, sequenced, and used for BLAST searches against the GenBank database (20). The presence of the *A. xylosoxidans* species-specific marker *blaOXA-114* was investigated by PCR amplification as described by Barrado et al. (6). After amplification, the PCR products were sequenced and compared with sequences in the GenBank database at the NCBI using BLAST.

### Inclusion and exclusion criteria

The respiratory secretion culture results of patients with CF were evaluated using the LBACT-UERJ database. The inclusion criteria were as follows: 1) patients with one or more cultures that were positive for *A. xylosoxidans* (the term “colonization/infection” is used in reference to positive cultures), and 2) patients who were colonized/infected with *A. xylosoxidans* and chronically colonized with *P. aeruginosa*, defined as more than 50% of cultures positive for the latter agent during 1 year (21). The exclusion criteria consisted of colonization with BCC bacteria and/or the absence of chronic colonization with *P. aeruginosa*.

### Definition of the groups

Patients were subdivided according to their *A. xylosoxidans* colonization/infection status into a chronically colonized/infected group and an intermittently colonized/infected group. The criterion for chronic colonization/infection by *A. xylosoxidans* was the same as that adopted for *P. aeruginosa* (21). Any shorter frequency was considered to be intermittent colonization/infection. The control group consisted of individuals who never had a positive culture for *A. xylosoxidans,* and subjects were matched with those in the case groups according to age (±1 year), sex, and chronic colonization with *P. aeruginosa*. All of the patients were chronically colonized by *P. aeruginosa*, and the status of *A. xylosoxidans* (chronic, intermittent, and never) was defined as described previously. Therefore, the three study groups were as follows: group I, chronic colonization/infection with *A. xylosoxidans* and chronic colonization with *P. aeruginosa*; group II, intermittent colonization/infection with *A. xylosoxidans* and chronic colonization with *P. aeruginosa*; and group III, never colonized/infected with *A. xylosoxidans,* but chronically colonized by *P. aeruginosa*.

### Clinical outcomes

The general population was described according to the demographic characteristics, diagnostic criteria for CF, and the presence of exocrine pancreatic insufficiency, cystic fibrosis-related diabetes, and liver disease. The frequency of the *F508del* mutation was described when available. In addition, other chronic bacterial co-colonizations were recorded by adopting the same criteria for chronic colonization as those used for *P. aeruginosa* (21).

Cross-sectional registration of clinical data was performed on two occasions: when the first positive culture for *A. xylosoxidans* occurred (moment 1 [M1]) and as close as possible to 24 months after the first positive culture (moment 2 [M2]). In the control group, data from M1 were paired with those of subjects in the case groups (groups II and III), and the same criteria were followed for M2.

With regard to lung function, the values of forced expiratory volume in 1 s (FEV_1_) and forced vital capacity (FVC) were recorded for all patients who were old enough to perform these tests. We recorded the best lung function value that was closest to the time of initial colonization. Similarly, we also recorded the best lung function that was obtained closest to 24 months later. The measurements were obtained with the HD CPL model (nSpire Health, Inc., USA) following the appropriate standards set by the American Thoracic Society (22). The pulmonary function results are reported as a percentage of the predicted values for the Brazilian population (23). The weight and height of patients were used to calculate body mass index (BMI). FEV_1_, FVC, and BMI were compared for the two time periods within and between groups. The median number of annual admissions was also compared between the groups.

### Statistical analysis

Numerical data are reported as means±SD or medians and ranges (minimum-maximum). Categorical data are reported as frequencies (%). The variables had a non-normal distribution according to the Kolmogorov-Smirnov test. Therefore, a non-parametric test was applied. Kruskal-Wallis ANOVA, with the corresponding Dunn's multiple comparison test, was used to compare numerical variables between the three groups. Fisher's exact test was used to compare categorical variables. When the association between categorical variables within the group was significant at 5%, Fisher's exact test, set for each peer group separately, was used. Therefore, we aimed to identify which groups differed from each other at a level of 1.7%. A level of 1.7% (5% divided by the number comparisons: 0.05/3=0.017) was used to control for type I error. To determine the existence of significant variations in FEV_1_, FVC, and BMI values between M1 and M2, the Wilcoxon signed rank test was used. Data analysis was performed using SAS software version 6.11 (SAS Institute, Inc., USA). The level of statistical significance was set at P<0.05.

## Results

Of the 238 individuals with culture results, 47 (19.7%) had at least one positive culture for *A. xylosoxidans*, among whom 25 met the inclusion criteria for the study. Ten patients were classified as chronically colonized/infected and 15 were classified as intermittently colonized/infected. The control group consisted of 18 patients. No participants died during the study period. The general characteristics of the study population and comparison between groups at baseline are reported in Table 1.

**Table:**
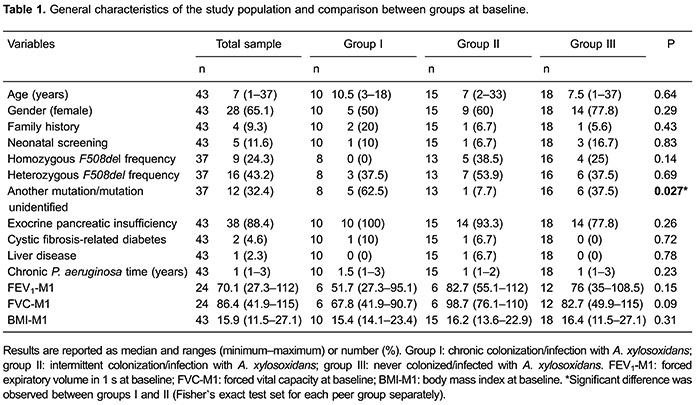

The median period of chronic colonization with *P. aeruginosa* was 1 year, and this ranged from 1 to 3 years. The baseline values for age, sex, *F508del* mutation frequency, exocrine pancreatic insufficiency, diabetes, liver disease, length of colonization with *P. aeruginosa*, and BMI were similar among the three groups. FEV_1_ and FVC values were lower in the chronically colonized/infected group, but this difference was not significant compared with the other groups (Table 1).

When the two periods (M1 and M2) were compared, there was a significant increase in FEV_1_ (P=0.014) and significant reduction in FVC (P=0.016) for the total sample. However, no significant changes were observed for these parameters for the patient groups. The median number of annual admissions during the study period was significantly different between the groups (P=0.033). There was a higher number of annual admissions in the chronically colonized/infected group compared to the never colonized/infected group. Information regarding pulmonary function, BMI, and clinical data for each group at M1 and M2 is reported in Table 2.

**Table:**
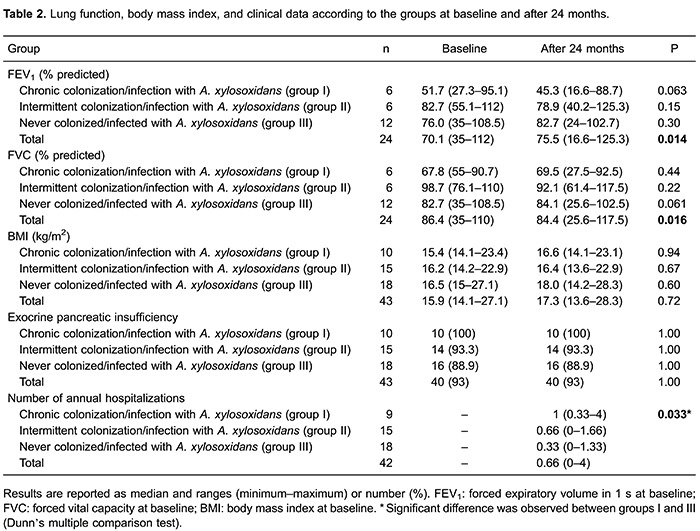

**Figure 1:**
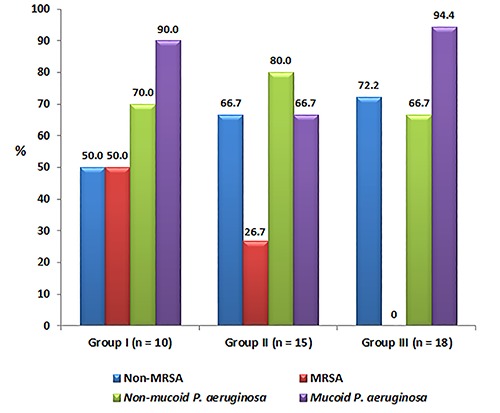
Distribution of chronic co-colonization according to the following groups: group I, chronic colonization/infection with *A. xylosoxidans* and chronic colonization with *P. aeruginosa*; group II, intermittent colonization/infection with *A. xylosoxidans* and chronic colonization with *P. aeruginosa*; and group III, never colonized/infected with *A. xylosoxidans,* but chronically colonized with *P. aeruginosa*. A significant difference in chronic co-colonization with methicillin-resistant *S. aureus* (MRSA) was found among the three groups (P=0.002; significant difference was observed between groups I and III using Fisher's exact test set for each peer group separately). No significant difference was observed for other types of chronic colonization.

Chronic co-colonization in each group is shown in Figure 1. A significant difference (P=0.002) in chronic co-colonization with methicillin-resistant *S. aureus* (MRSA) among the three groups was observed. Chronic co-colonization with MRSA was observed in 50% and 26.7% of the patients who were chronically colonized/infected with *A. xylosoxidans* and intermittently colonized/infected with *A. xylosoxidans*, respectively. No patients without colonization/infection with *A. xylosoxidans* had chronic co-colonization with MRSA. No significant difference was observed for other types of chronic colonization.

## Discussion

The main findings of this study were as follows: in CF patients, a relatively high frequency of *A. xylosoxidans* colonization/infection was present among children, and reduced lung function in patients who were chronically colonized/infected with *A. xylosoxidans* was observed. In addition, we did not observe any differences in clinical endpoints over 2 years when we compared patients who were chronically colonized with *P. aeruginosa*, with or without *A. xylosoxidans,* except for an increased number of hospital admissions for patients with *A. xylosoxidans*.

In the present study, the frequency of colonization/infection with *A. xylosoxidans* (19.7%) was similar to the upper limit of the range reported in other studies (2% to 17.9%) (7,8). This reported frequency in our study was cumulative, which explains the higher percentage than other studies. The large range in *A. xylosoxidans* colonization/infection frequency may be partly attributed to methodological differences between the studies (6 7-8,11,12). The prevalence of colonization/infection with *A. xylosoxidans* in pediatric patients in our study (median, 7 years) was different from that reported in other studies, which showed that it was predominantly observed during late adolescence or early adulthood (10,12 13-14). The most similar results to our study are those of a French study that reported a median age of 10.3 years (variation of 6 to 14 years) for the first positive culture among patients with CF who became chronically colonized/infected with *A. xylosoxidans* (24). However, notably, this French study only included children and adolescents.

There is no universal criterion for the definition of chronic colonization with *A. xylosoxidans* (6 7-8,11 12-13). The criterion of Pereira et al. (25) is more consistent for ensuring chronicity. However, the criterion of chronicity that was adopted in the present study (21) included an assumption that patient care needs were satisfied. Therefore, clinical measures must be adopted during the short period in which colonization can harm the patient. One of the suspected risk factors for colonization/infection with *A. xylosoxidans* is treatment for *P. aeruginosa* (11). In our study, of the 47 patients who had at least one positive culture for *A. xylosoxidans*, 41 were colonized/infected with *P. aeruginosa*. However, only 25 met the criteria for chronic colonization/infection with *P. aeruginosa* without BCC colonization. All six patients in whom *P. aeruginosa* was not identified were colonized with BCC. Despite the restriction of the sample size by the selection criteria, chronic colonization with *P. aeruginosa* was considered important for subject pairing because it decreased the chance of bias in the outcomes of interest.

Interestingly, none of the patients in the group of patients who were chronically colonized/infected with *A*. *xylosoxidans* were homozygous for the *F508del* mutation, and its frequency was smaller than that found in the other groups. Therefore, other serious mutations may be more frequent in this population, as suggested by Cabello et al. (26). With regard to lung function, FEV_1_ values in the group of patients who were chronically colonized/infected with *A*. *xylosoxidans* were lower than those found in the other two groups. Although this finding was not statistically significant, clinically, this difference suggests a more advanced stage of lung damage among individuals who became chronically colonized/infected with *A*. *xylosoxidans*. These data are consistent with the hypothesis proposed by De Baets et al. (8), in which individuals with increased lung impairment appear to be more prone to chronic colonization/infection with *A*. *xylosoxidans*.

A higher frequency of hospitalizations and chronic co-colonization with MRSA was observed in the group of patients who were colonized/infected with *A*. *xylosoxidans,* and this frequency was highest in the chronically colonized/infected group. Zemanick et al. (27) found a higher number of exacerbations requiring intravenous treatment and a higher relative risk of isolation of MRSA, *S. maltophilia*, and *A. xylosoxidans* after the first isolation of *P. aeruginosa*. A recent multicenter study showed that the frequency of colonization with MRSA has increased in recent years (28). Additionally, colonization with *P. aeruginosa* and more intensive therapeutic interventions may be risk factors for chronic colonization with MRSA, particularly for healthcare-associated MRSA (HA-MRSA) (28). In our study, although chronic colonization with *P. aeruginosa* was a criterion for pairing, MRSA was not found in the group that was not colonized/infected with *A. xylosoxidans*.

Compared with the other two groups, there was higher number of hospitalizations in the chronically colonized/infected group. This finding may be explained by the fact that the condition of this group of patients was more severe at the beginning of the study or because other conditions contributed to this outcome. Notably, we were unable to determine whether the association between chronic colonization with MRSA and *A. xylosoxidans* was the result of increased hospitalization and more intensive antimicrobial therapy, or whether any real association existed between these two agents or between these agents and mutations, as previously reported for *P. aeruginosa* (29).

The chronically colonized/infected group showed much smaller FEV_1_ values than the intermittently colonized/infected and non-colonized/infected groups, at the time of colonization/infection and approximately 2 years later. Similarly, other studies have shown a higher frequency of colonization/infection with *A. xylosoxidans* in individuals with CF with more severe lung disease (13). In the present study, no significant difference in intra- or inter-group variation was observed for these parameters. Nevertheless, over 2 years, FEV_1_ values decreased in the chronically colonized/infected group by 6.4% of the predicted value and by 3.8% in the intermittently colonized/infected group (Table 2). Interestingly, Llorca Otero et al. (30) observed a mean annual decline in FEV_1_ of 2.49% in patients who were chronically colonized/infected with *A. xylosoxidans*.

A strength of the current study is that it is the first Brazilian study to determine a relationship between clinical data and colonization/infection with *A. xylosoxidans*. However, the present study has major limitations. First, our study was limited by the broad age range and small sample size. These factors can, at least in part, be explained by the study's retrospective design and the fact that *A. xylosoxidans* has a low incidence/prevalence in CF patients. Second, our population was exclusively composed of patients who were chronically colonized with *P. aeruginosa*. However, treatment for chronic colonization with *P. aeruginosa* might favor the emergence of *A. xylosoxidans* (10,11). Notwithstanding these limitations, this study can serve as a starting point for future clinical trials to evaluate intervention protocols in CF patients who are colonized/infected with *A. xylosoxidans*.

In conclusion, a relatively high frequency of *A. xylosoxidans* colonization/infection was present in children; and reduced lung function was observed in patients who were chronically colonized/infected with *A. xylosoxidans*. This colonized/infected group also showed an increased frequency of chronic colonization with MRSA. In addition, no significant differences in clinical endpoints were observed over 2 years, except for an increased number of hospitalizations in patients with *A. xylosoxidans*. With regard to the change in lung function over 2 years, a trend toward a decrease in FEV_1_ values of patients who were chronically colonized/infected with *A. xylosoxidans* was observed.
